# Tetra­kis(μ_3_-2-{[1,1-bis­(hy­droxy­meth­yl)-2-oxidoeth­yl]imino­meth­yl}-6-nitro­pheno­lato)tetra­copper(II)

**DOI:** 10.1107/S1600536814000798

**Published:** 2014-01-18

**Authors:** Eduard N. Chygorin, Yuri O. Smal, Vladimir N. Kokozay, Irina V. Omelchenko

**Affiliations:** a Department of Inorganic Chemistry, Taras Shevchenko National University of Kyiv, 64\13 Volodymyrska St, Kyiv 01601, Ukraine; b STC ‘Institute for Single Crystals’, National Academy of Sciences of Ukraine, 60 Lenina Avenue, Kharkiv 61001, Ukraine

## Abstract

The title cluster, [Cu_4_(C_11_H_12_N_2_O_6_)_4_], was obtained from the Cu^0^–FeCl_2_·4H_2_O–H_4_ *L*–Et_3_N–DMF reaction system (in air), where H_4_ *L* is 2-hy­droxy­methyl-2{[(2-hy­droxy-3-nitro­phen­yl)methyl­idene]amino}­propane-1,3-diol and DMF is di­methyl­formamide. The asymmetric unit consists of one Cu^2+^ ion and one dianionic ligand; a -4 symmetry element generates the cluster, which contains a {Cu_4_O_4_} cubane-like core. The metal ion has an elongated square-based pyramidal CuNO_4_ coordination geometry with the N atom in a basal site. An intra­molecular O—H⋯O hydrogen bond is observed. The solvent mol­ecules were found to be highly disordered and their contribution to the scattering was removed with the SQUEEZE procedure in *PLATON* [Spek (2009 ▶). *Acta Cryst*. D**65**, 148–155], which indicated a solvent cavity of volume 3131 Å^3^ containing approximately 749 electrons. These solvent molecules are not considered in the given chemical formula.

## Related literature   

For general background to direct synthesis (DS), see: Kokozay & Shevchenko (2005 ▶). For related structures, see: Dey *et al.* (2002 ▶); Dong *et al.* (2007 ▶); Guo *et al.* (2008 ▶). For successful realisation of DS, see: Chygorin *et al.* (2012 ▶); Nesterov *et al.* (2012 ▶).
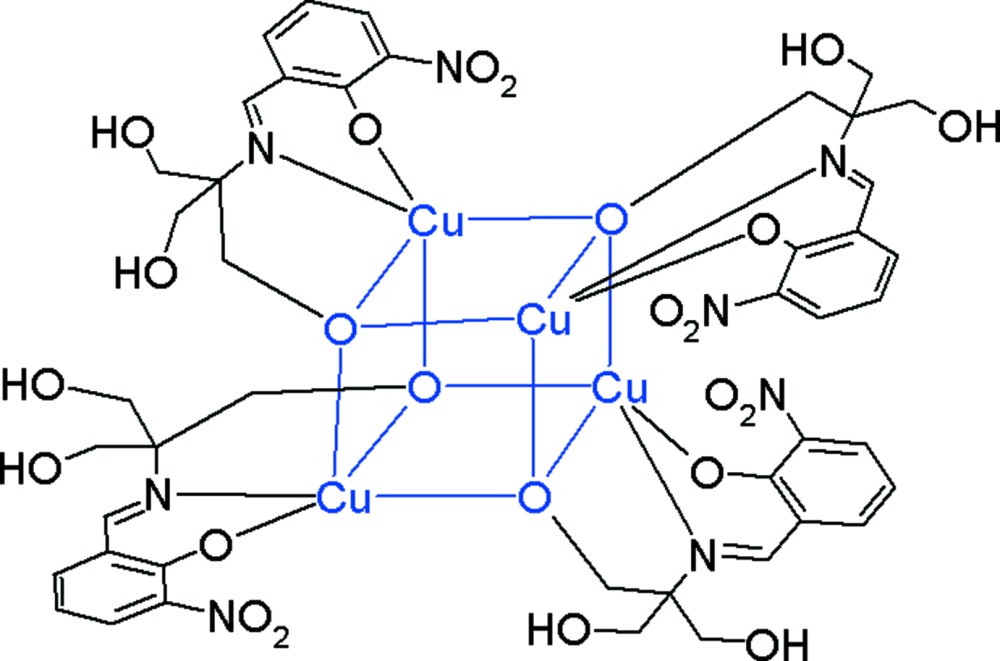


## Experimental   

### 

#### Crystal data  

[Cu_4_(C_11_H_12_N_2_O_6_)_4_]
*M* *_r_* = 1327.06
Tetragonal, 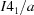

*a* = 20.5587 (14) Å
*c* = 18.010 (2) Å
*V* = 7612.0 (11) Å^3^
*Z* = 4
Mo *K*α radiation
μ = 1.17 mm^−1^
*T* = 173 K
0.40 × 0.40 × 0.30 mm

#### Data collection  

Agilent Xcalibur Sapphire3 diffractometer
Absorption correction: multi-scan (*CrysAlis PRO*; Agilent, 2012) ▶ *T*_min_ = 0.653, *T*_max_ = 0.721
3349 measured reflections
3349 independent reflections
1395 reflections with *I* > 2σ(*I*)

#### Refinement  

*R*[*F* ^2^ > 2σ(*F* ^2^)] = 0.075
*wR*(*F* ^2^) = 0.184
*S* = 0.80
3349 reflections
182 parameters
H-atom parameters constrained
Δρ_max_ = 0.94 e Å^−3^
Δρ_min_ = −0.57 e Å^−3^

### 

Data collection: *CrysAlis CCD* (Agilent, 2012 ▶); cell refinement: *CrysAlis RED* (Agilent, 2012 ▶); data reduction: *CrysAlis RED*; program(s) used to solve structure: *SHELXTL* (Sheldrick, 2008 ▶); program(s) used to refine structure: *OLEX2* (Dolomanov *et al.*, 2009 ▶); molecular graphics: *SHELXTL*; software used to prepare material for publication: *publCIF* (Westrip, 2010 ▶).

## Supplementary Material

Crystal structure: contains datablock(s) I, New_Global_Publ_Block. DOI: 10.1107/S1600536814000798/hb7174sup1.cif

Structure factors: contains datablock(s) I. DOI: 10.1107/S1600536814000798/hb7174Isup2.hkl

CCDC reference:

Additional supporting information:  crystallographic information; 3D view; checkCIF report

## Figures and Tables

**Table 1:** Selected bond lengths (Å)

Cu1—O1	1.892 (5)
Cu1—O6^i^	1.940 (5)
Cu1—N1	1.952 (6)
Cu1—O6	1.954 (4)
Cu1—O6^ii^	2.524 (5)

Symmetry codes: (i) 
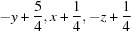
; (ii) 
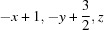
.

**Table 2:** Hydrogen-bond geometry (Å, °)

*D*—H⋯*A*	*D*—H	H⋯*A*	*D*⋯*A*	*D*—H⋯*A*
O4—H4*A*⋯O4^ii^	0.78	1.96	2.729 (9)	171

Symmetry code: (ii) 
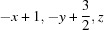
.

**Fig. 1.**
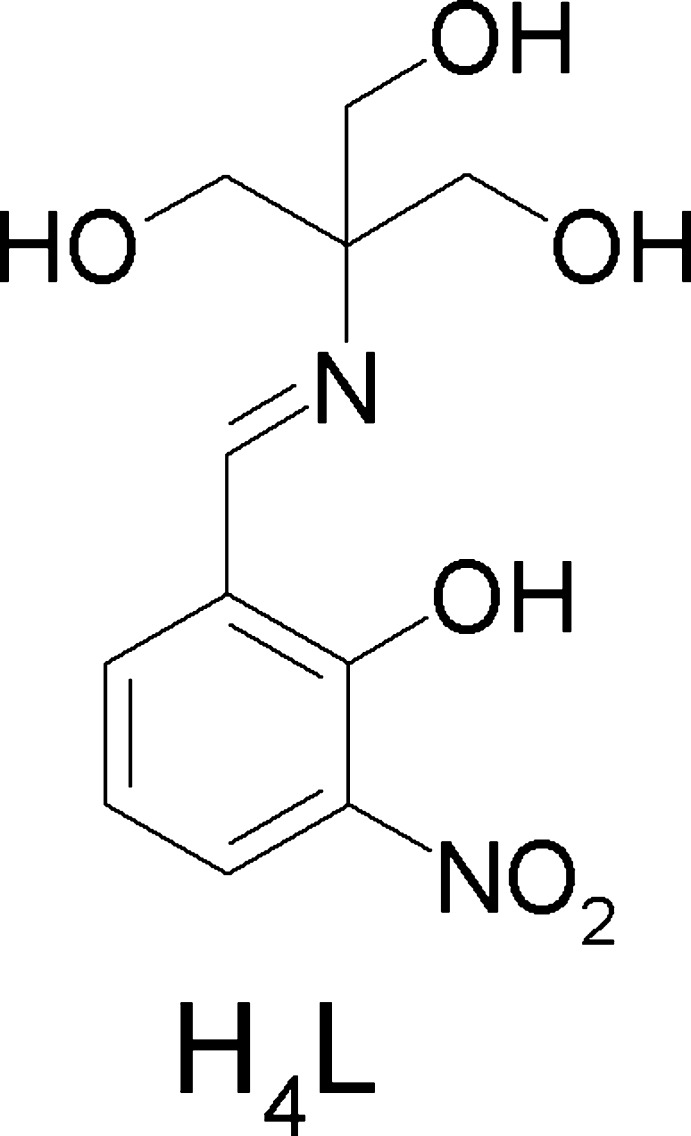
Schiff base ligand: the product of condensation of 3-nitro-salicylaldehyde and tris(hydroxymethyl)aminomethane.

**Fig. 2.**
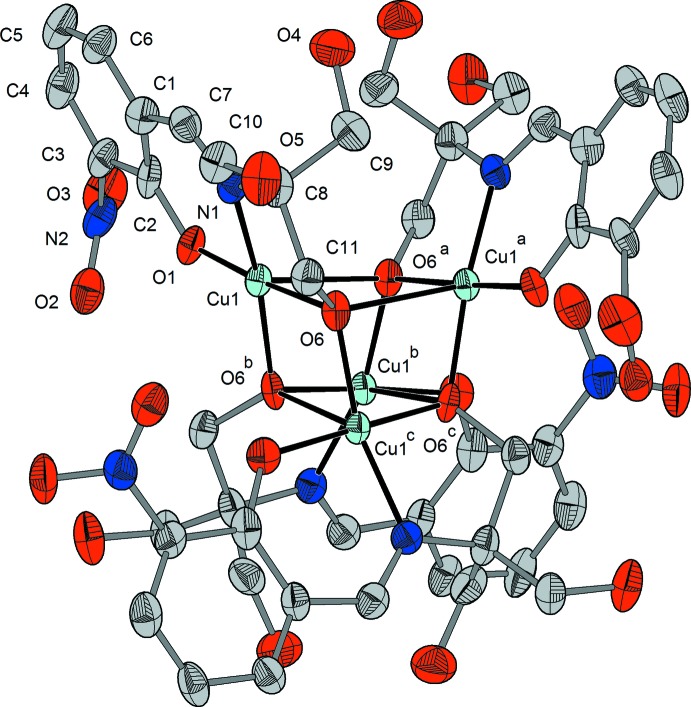
View of cubane tetranuclear complex [Cu4(C11H12O6N2)4] (H atoms are omitted for clarity, the non-hydrogen atoms are shown as 30% thermal ellipsoids). Symmetry transformation used to generate equivalent atoms: a 1 - x, 1.5 - y, z; b x + 0.25, 1.25 - y, 0.25 - z; c 1.25 - x, y - 0.25,0.25 - z.

**Fig. 3.**
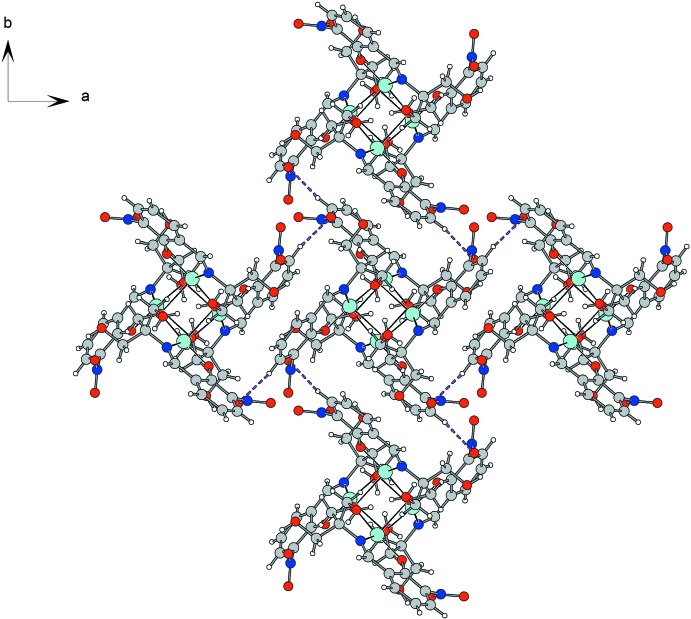
The crystal-packing diagram along the (001) direction.

## supplementary crystallographic information

### 1. Comment

In last few decades polynuclear complexes have been in focus of intense interest due to their relevance to the active sites of metaloenzimes, and their potential applications as magnetic materials. Thus development of synthetic approaches that could lead to new polynuclear compounds or improve their yields is quite important. Our research group is interested in employment of so-called "direct synthesis" (DS), a serendipitous self-assembling approach based on utilization of metal powders as starting materials to construct coordination compounds both homo- and heterometallic ones. Recently we have shown its ability to produce Co/Fe complexes with Schiff base ligand (Chygorin *et al.*, 2012; Nesterov *et al.*, 2012). It should be noted that outcome of DS is not highly predictable and sometimes we can isolated homometallic or mononuclear complexes only. Such a case was observed in the investigated system: Cu^0^–FeCl_2_^.^4H_2_O–H_4_L–Et_3_N–dmf, where H_4_L is 2-hydroxymethyl-2{[(2-hydroxy-3-nitrophenyl)methylene]amino}propane-1,3-diol (Fig. 1). The Schiff base ligand, that is obtained by condencation of the salicylaldehyde derivative and tris(hydroxymethyl)aminomethane is typical hydroxy-rich ligand, which can coordinate to several metal centers and accepts various coordination modes, and thus it is an attractive ligand system for serendipitous self-assembling. Despite of this fact this Schiff base ligand has recived little attention to date [only 35 hits were found by searching *via* CSD (http://www.ccdc.cam.ac.uk/cgi-bin/catreq.cgi?)]. Herein we report the synthesis of a new tetranuclear cubane complex starting from potentially polydentate hydroxyl-rich ligand.

The reaction of copper powder with iron(II) chloride in dmf solution of the tetrapodal Schiff base ligand, formed *in situ*, in basic medium with free access of air leads to the isolation of the homometallic cuban complex [Cu_4_(C_11_H_12_O_6_N_2_)_4_]. The Schiff base ligand H_4_L was obtained by condensation of 3-nitro-salicylaldehyde and tris(hydroxymethyl)aminomethane (Fig. 1). The molar ratio of starting materials (Cu^0^: FeCl_2_: Schiff base ligand) was taken 1:1:2. The reaction was carried out in air with heating and stirring till total dissolution of metal powder was observed.

Tetranuclear molecular complex (Fig. 2) consists of the discrete [Cu_4_(H_2_L)_4_] moiety with a *{Cu_4_O_4_}* cubane-like core. Eight alternately arranged netal centers and oxygen atoms from methoxy groups form a distorted *{Cu_4_O_4_}* cube with local *S_4_*-symmetry. Each of four ligands coordinates in a tridentate mode as an (H_2_L)^2-^ dianion, with the phenoxyl and one of the alkoxyl groups deprotonated. The NO_2_ donor set from one ligand molecule together with O-atom from methoxy arm of another ligand forms distorted square coordination polyhedra around each metal center (with RMS deviation of atoms from square plane of 0.135 Å). Coordination lengths vary in the range of 1.892 - 1.955 Å, and *X*—Cu—Y angles vary in the range of 84.8 - 94.9° that is comparable with the known literature data. The oxygen atom of the methoxy group of the third ligand molecule coordinates on this metal atom with Cu—O length of 2.524 Å, so that can be threated as additional coordination. In crystal, weak C4—H4B···O2' hydrogen bonds (1.25 - *y*,*x* - 0.25,*z* - 0.25; H···O' 2.51 Å, C—H···O' 153°) form three-dimensional-connected network with channels along (111) crystallographic direction. Minimal channel dimension is about 6.74 Å (O5···O5' distance). The crystal packing diagram is shown in Fig. 3.

### 2. Experimental

Tris(hydroxymethyl)aminomethane (0.303 g, 2.5 mmol), 3-Nitrosalicylaldehyde (0.418 g, 2.5 mmol), and triethylamine (0.35 ml, 2.5 mmol) were dissolved in dmf (25 ml) in this order, forming an orange solution and magnetically stirred at 60–70°C (15 min). Then, copper powder (0.079 g, 1.25 mmol) and FeCl_2_^.^4H_2_O (0.248 g, 1.25 mmol) were successfully added to the hot orange solution with stirring about 3 h. Brown blocks were isolated by adding diethylether to the dark orange-brown solution after 2 days. Yield: 0.4 g, 48%. The compound is sparingly soluble in dmso and dmf, and it is stable in air.

### 3. Refinement

All H atoms were placed in idealized positions (C–H = 0.95 – 0.99 Å, O–H = 0.84 Å) and constrained to ride on their parent atoms, with *U*_iso_ = 1.2Ueq (except *U*_iso_ = 1.5Ueq for hydroxyl groups). Hydrogen atom of the hydroxyl group O4–H4 was disordered over two sites with equal occupancy factors of 0.50 in order to fit the intramolecular hydrogen bond O4–H4A···O4'. Several isolated electron density peaks were located during the refinement, whose were believe to be a solvent molecules. Large displacement parameters were observed modeling the disordered oxygen, carbon, and sulfur atoms. SQUEEZE procedure of *PLATON* indicated a solvent cavity of volume 3131 Å^3^ centered at (0,0,0), containing approximately 749 electrons. In the final refinement, this contribution was removed from the intensity data that produced better refinement results. The hydroxyl group O5—H5A located near the void was believed to be H-bonded with one of the removed solvent molecules. Several reflections with great differences between calculated and observed F^2^ were omitted during the refinement. These reflections were believed to arise because of little impurities of the crystal under study.

### Crystal data

**Table:**

[Cu_4_(C_11_H_12_N_2_O_6_)_4_]	*D*_x_ = 1.158 Mg m^−^^3^
*M**_r_* = 1327.06	Mo *K*α radiation, λ = 0.71073 Å
Tetragonal, *I*4_1_/*a*	Cell parameters from 462 reflections
Hall symbol: -I 4ad	θ = 3.0–25.0°
*a* = 20.5587 (14) Å	µ = 1.17 mm^−^^1^
*c* = 18.010 (2) Å	*T* = 173 K
*V* = 7612.0 (11) Å^3^	Block, brown
*Z* = 4	0.40 × 0.40 × 0.30 mm
*F*(000) = 2704	

### Data collection

**Table:**

Agilent Xcalibur Sapphire3 diffractometer	3349 independent reflections
Radiation source: Enhance (Mo) X-ray Source	1395 reflections with *I* > 2σ(*I*)
Graphite monochromator	*R*_int_ = 0.000
Detector resolution: 16.1827 pixels mm^-1^	θ_max_ = 25.0°, θ_min_ = 3.2°
ω scans	*h* = −16→17
Absorption correction: multi-scan (*CrysAlis PRO*; Agilent, 2012)	*k* = 0→24
*T*_min_ = 0.653, *T*_max_ = 0.721	*l* = 0→21
3349 measured reflections	

### Refinement

**Table:**

Refinement on *F*^2^	Primary atom site location: structure-invariant direct methods
Least-squares matrix: full	Secondary atom site location: difference Fourier map
*R*[*F*^2^ > 2σ(*F*^2^)] = 0.075	Hydrogen site location: inferred from neighbouring sites
*wR*(*F*^2^) = 0.184	H-atom parameters constrained
*S* = 0.80	*w* = 1/[σ^2^(*F*_o_^2^) + (0.060*P*)^2^] where *P* = (*F*_o_^2^ + 2*F*_c_^2^)/3
3349 reflections	(Δ/σ)_max_ = 0.001
182 parameters	Δρ_max_ = 0.94 e Å^−^^3^
0 restraints	Δρ_min_ = −0.57 e Å^−^^3^

### Special details

**Table:**

Geometry. All e.s.d.'s (except the e.s.d. in the dihedral angle between two l.s. planes) are estimated using the full covariance matrix. The cell e.s.d.'s are taken into account individually in the estimation of e.s.d.'s in distances, angles and torsion angles; correlations between e.s.d.'s in cell parameters are only used when they are defined by crystal symmetry. An approximate (isotropic) treatment of cell e.s.d.'s is used for estimating e.s.d.'s involving l.s. planes.
Refinement. Refinement of *F*^2^ against ALL reflections. The weighted *R*-factor *wR* and goodness of fit *S* are based on *F*^2^, conventional *R*-factors *R* are based on *F*, with *F* set to zero for negative *F*^2^. The threshold expression of *F*^2^ > σ(*F*^2^) is used only for calculating *R*-factors(gt) *etc*. and is not relevant to the choice of reflections for refinement. *R*-factors based on *F*^2^ are statistically about twice as large as those based on *F*, and *R*- factors based on ALL data will be even larger.

### Fractional atomic coordinates and isotropic or equivalent isotropic displacement parameters (Å^2^)

**Table:**

	*x*	*y*	*z*	*U*_iso_*/*U*_eq_	Occ. (<1)
Cu1	0.48941 (4)	0.66864 (4)	0.06731 (4)	0.0396 (3)	
O1	0.5536 (2)	0.6041 (2)	0.0524 (3)	0.0451 (13)	
N1	0.4460 (3)	0.6539 (3)	−0.0276 (3)	0.0406 (16)	
C1	0.5204 (4)	0.5693 (4)	−0.0716 (4)	0.047 (2)	
N2	0.6541 (4)	0.5153 (3)	0.0538 (4)	0.0542 (19)	
O2	0.6303 (3)	0.5101 (3)	0.1159 (3)	0.0611 (17)	
C2	0.5608 (4)	0.5690 (4)	−0.0063 (5)	0.047 (2)	
O3	0.7144 (3)	0.5100 (3)	0.0436 (3)	0.0751 (19)	
C3	0.6141 (4)	0.5230 (4)	−0.0096 (5)	0.050 (2)	
O4	0.4358 (3)	0.7332 (3)	−0.1606 (3)	0.0761 (19)	
H4A	0.4728	0.7401	−0.1646	0.114*	0.50
H4C	0.4145	0.7014	−0.1769	0.114*	0.50
C4	0.6276 (4)	0.4875 (4)	−0.0723 (5)	0.061 (2)	
H4B	0.6644	0.4596	−0.0728	0.073*	
O5	0.2776 (3)	0.7029 (3)	−0.0736 (4)	0.0732 (18)	
H5A	0.2430	0.6816	−0.0784	0.110*	
C5	0.5890 (4)	0.4913 (4)	−0.1342 (5)	0.061 (3)	
H5B	0.5991	0.4672	−0.1777	0.073*	
O6	0.4282 (2)	0.7408 (2)	0.0779 (2)	0.0369 (12)	
C6	0.5353 (4)	0.5310 (4)	−0.1317 (4)	0.052 (2)	
H6A	0.5071	0.5320	−0.1735	0.063*	
C7	0.4659 (4)	0.6122 (4)	−0.0773 (4)	0.048 (2)	
H7A	0.4416	0.6101	−0.1220	0.057*	
C8	0.3906 (4)	0.6972 (4)	−0.0412 (4)	0.047 (2)	
C9	0.4121 (4)	0.7530 (4)	−0.0911 (4)	0.057 (2)	
H9A	0.3747	0.7826	−0.0989	0.069*	
H9B	0.4465	0.7780	−0.0653	0.069*	
C10	0.3319 (4)	0.6594 (4)	−0.0737 (5)	0.058 (2)	
H10A	0.3415	0.6448	−0.1249	0.069*	
H10B	0.3223	0.6206	−0.0430	0.069*	
C11	0.3712 (4)	0.7262 (4)	0.0359 (4)	0.0436 (19)	
H11A	0.3454	0.7663	0.0285	0.052*	
H11B	0.3440	0.6945	0.0633	0.052*	

### Atomic displacement parameters (Å^2^)

**Table:**

	*U*^11^	*U*^22^	*U*^33^	*U*^12^	*U*^13^	*U*^23^
Cu1	0.0337 (6)	0.0344 (6)	0.0509 (5)	0.0009 (5)	−0.0021 (5)	−0.0018 (4)
O1	0.044 (3)	0.029 (3)	0.062 (3)	−0.005 (3)	−0.002 (3)	−0.015 (3)
N1	0.039 (4)	0.037 (4)	0.046 (3)	−0.005 (3)	−0.001 (3)	−0.006 (3)
C1	0.032 (5)	0.046 (5)	0.064 (5)	0.001 (4)	0.007 (4)	−0.004 (5)
N2	0.053 (5)	0.038 (4)	0.071 (5)	0.012 (4)	−0.004 (4)	−0.017 (4)
O2	0.059 (4)	0.040 (4)	0.084 (4)	0.008 (3)	−0.019 (4)	−0.004 (3)
C2	0.034 (5)	0.022 (4)	0.084 (6)	−0.001 (4)	0.003 (5)	−0.009 (4)
O3	0.032 (4)	0.077 (5)	0.116 (5)	0.013 (3)	−0.001 (3)	−0.026 (4)
C3	0.039 (5)	0.041 (5)	0.071 (5)	−0.023 (4)	−0.002 (5)	−0.018 (5)
O4	0.082 (5)	0.088 (5)	0.059 (3)	−0.014 (4)	0.006 (3)	0.006 (3)
C4	0.039 (5)	0.045 (6)	0.098 (6)	−0.004 (5)	0.005 (5)	−0.024 (5)
O5	0.038 (4)	0.065 (4)	0.116 (5)	0.001 (3)	−0.020 (4)	0.000 (4)
C5	0.037 (5)	0.051 (6)	0.094 (6)	−0.004 (5)	0.020 (5)	−0.033 (5)
O6	0.019 (3)	0.029 (3)	0.063 (3)	0.002 (2)	−0.007 (2)	0.004 (2)
C6	0.040 (5)	0.054 (6)	0.062 (5)	−0.012 (5)	0.005 (4)	−0.016 (5)
C7	0.044 (5)	0.048 (5)	0.051 (5)	−0.013 (4)	−0.001 (4)	0.010 (4)
C8	0.032 (5)	0.043 (5)	0.067 (5)	0.005 (4)	−0.013 (4)	0.004 (4)
C9	0.048 (6)	0.063 (6)	0.062 (5)	−0.010 (5)	−0.017 (4)	0.010 (5)
C10	0.041 (5)	0.061 (6)	0.071 (5)	0.005 (5)	0.000 (5)	−0.007 (5)
C11	0.030 (5)	0.041 (5)	0.059 (4)	0.000 (4)	0.003 (4)	−0.002 (4)

### Geometric parameters (Å, º)

**Table:**

Cu1—O1	1.892 (5)	C4—C5	1.371 (11)
Cu1—O6^i^	1.940 (5)	C4—H4B	0.9500
Cu1—N1	1.952 (6)	O5—C10	1.430 (9)
Cu1—O6	1.954 (4)	O5—H5A	0.8400
Cu1—O6^ii^	2.524 (5)	C5—C6	1.374 (11)
O1—C2	1.288 (8)	C5—H5B	0.9500
N1—C7	1.304 (9)	O6—C11	1.428 (8)
N1—C8	1.467 (9)	O6—Cu1^iii^	1.940 (5)
C1—C6	1.373 (10)	C6—H6A	0.9500
C1—C7	1.428 (10)	C7—H7A	0.9500
C1—C2	1.440 (10)	C8—C9	1.524 (10)
N2—O2	1.224 (8)	C8—C10	1.551 (10)
N2—O3	1.259 (8)	C8—C11	1.562 (10)
N2—C3	1.417 (10)	C9—H9A	0.9900
C2—C3	1.449 (10)	C9—H9B	0.9900
C3—C4	1.373 (10)	C10—H10A	0.9900
O4—C9	1.403 (9)	C10—H10B	0.9900
O4—H4A	0.7773	C11—H11A	0.9900
O4—H4C	0.8400	C11—H11B	0.9900
			
O1—Cu1—O6^i^	93.6 (2)	C6—C5—H5B	120.9
O1—Cu1—N1	94.9 (2)	C11—O6—Cu1^iii^	118.5 (4)
O6^i^—Cu1—N1	164.4 (2)	C11—O6—Cu1	108.5 (4)
O1—Cu1—O6	174.8 (2)	Cu1^iii^—O6—Cu1	108.6 (2)
O6^i^—Cu1—O6	87.9 (2)	C1—C6—C5	123.1 (8)
O1—Cu1—O6^ii^	93.45 (18)	C1—C6—H6A	118.5
O6^i^—Cu1—O6^ii^	73.19 (17)	C5—C6—H6A	118.5
N1—Cu1—O6^ii^	119.2 (2)	N1—C7—C1	127.0 (7)
O6—Cu1—O6^ii^	82.22 (17)	N1—C7—H7A	116.5
N1—Cu1—O6	84.8 (2)	C1—C7—H7A	116.5
C2—O1—Cu1	126.0 (5)	N1—C8—C9	109.3 (6)
C7—N1—C8	121.9 (6)	N1—C8—C10	111.3 (6)
C7—N1—Cu1	124.0 (5)	C9—C8—C10	112.3 (6)
C8—N1—Cu1	114.0 (5)	N1—C8—C11	106.4 (6)
C6—C1—C7	118.2 (8)	C9—C8—C11	108.1 (7)
C6—C1—C2	120.9 (8)	C10—C8—C11	109.2 (6)
C7—C1—C2	120.8 (7)	O4—C9—C8	114.1 (7)
O2—N2—O3	121.3 (7)	O4—C9—H9A	108.7
O2—N2—C3	120.9 (7)	C8—C9—H9A	108.7
O3—N2—C3	117.6 (7)	O4—C9—H9B	108.7
O1—C2—C1	127.1 (7)	C8—C9—H9B	108.7
O1—C2—C3	119.0 (7)	H9A—C9—H9B	107.6
C1—C2—C3	113.9 (7)	O5—C10—C8	107.0 (6)
C4—C3—N2	119.1 (8)	O5—C10—H10A	110.3
C4—C3—C2	122.3 (8)	C8—C10—H10A	110.3
N2—C3—C2	118.6 (7)	O5—C10—H10B	110.3
C9—O4—H4A	111.7	C8—C10—H10B	110.3
C9—O4—H4C	110.9	H10A—C10—H10B	108.6
H4A—O4—H4C	128.4	O6—C11—C8	110.0 (6)
C5—C4—C3	121.4 (8)	O6—C11—H11A	109.7
C5—C4—H4B	119.3	C8—C11—H11A	109.7
C3—C4—H4B	119.3	O6—C11—H11B	109.7
C10—O5—H5A	109.5	C8—C11—H11B	109.7
C4—C5—C6	118.2 (8)	H11A—C11—H11B	108.2
C4—C5—H5B	120.9		
			
O6^i^—Cu1—O1—C2	170.0 (6)	O6^i^—Cu1—O6—C11	−139.6 (4)
N1—Cu1—O1—C2	3.0 (6)	N1—Cu1—O6—C11	26.7 (4)
O6—Cu1—O1—C2	−84 (2)	O1—Cu1—O6—Cu1^iii^	−116 (2)
O6^ii^—Cu1—O1—C2	−116.7 (6)	O6^i^—Cu1—O6—Cu1^iii^	−9.5 (2)
O1—Cu1—N1—C7	−2.5 (6)	N1—Cu1—O6—Cu1^iii^	156.7 (3)
O6^i^—Cu1—N1—C7	−125.4 (8)	C7—C1—C6—C5	175.5 (7)
O6—Cu1—N1—C7	172.3 (6)	C2—C1—C6—C5	−0.9 (12)
O6^ii^—Cu1—N1—C7	94.2 (6)	C4—C5—C6—C1	3.4 (13)
O1—Cu1—N1—C8	−178.6 (5)	C8—N1—C7—C1	177.6 (7)
O6^i^—Cu1—N1—C8	58.5 (11)	Cu1—N1—C7—C1	1.8 (11)
O6—Cu1—N1—C8	−3.8 (5)	C6—C1—C7—N1	−177.0 (7)
O6^ii^—Cu1—N1—C8	−81.9 (5)	C2—C1—C7—N1	−0.6 (12)
Cu1—O1—C2—C1	−2.8 (11)	C7—N1—C8—C9	−77.3 (8)
Cu1—O1—C2—C3	178.0 (5)	Cu1—N1—C8—C9	98.9 (6)
C6—C1—C2—O1	177.4 (7)	C7—N1—C8—C10	47.3 (9)
C7—C1—C2—O1	1.0 (12)	Cu1—N1—C8—C10	−136.5 (5)
C6—C1—C2—C3	−3.4 (11)	C7—N1—C8—C11	166.2 (6)
C7—C1—C2—C3	−179.7 (7)	Cu1—N1—C8—C11	−17.6 (7)
O2—N2—C3—C4	−135.5 (8)	N1—C8—C9—O4	59.8 (8)
O3—N2—C3—C4	40.2 (11)	C10—C8—C9—O4	−64.2 (9)
O2—N2—C3—C2	45.5 (10)	C11—C8—C9—O4	175.3 (6)
O3—N2—C3—C2	−138.8 (8)	N1—C8—C10—O5	170.8 (6)
O1—C2—C3—C4	−175.1 (7)	C9—C8—C10—O5	−66.3 (8)
C1—C2—C3—C4	5.5 (11)	C11—C8—C10—O5	53.6 (8)
O1—C2—C3—N2	3.8 (11)	Cu1^iii^—O6—C11—C8	−167.6 (4)
C1—C2—C3—N2	−175.5 (7)	Cu1—O6—C11—C8	−43.2 (6)
N2—C3—C4—C5	177.6 (8)	N1—C8—C11—O6	39.5 (8)
C2—C3—C4—C5	−3.5 (13)	C9—C8—C11—O6	−77.8 (7)
C3—C4—C5—C6	−1.2 (13)	C10—C8—C11—O6	159.8 (6)
O1—Cu1—O6—C11	114 (2)		

Symmetry codes: (i) −*y*+5/4, *x*+1/4, −*z*+1/4; (ii) −*x*+1, −*y*+3/2, *z*; (iii) *y*−1/4, −*x*+5/4, −*z*+1/4.

### Hydrogen-bond geometry (Å, º)

**Table:**

*D*—H···*A*	*D*—H	H···*A*	*D*···*A*	*D*—H···*A*
O4—H4*A*···O4^ii^	0.78	1.96	2.729 (9)	171

Symmetry code: (ii) −*x*+1, −*y*+3/2, *z*.
